# Neural Radiance Fields for High-Fidelity Soft Tissue Reconstruction in Endoscopy

**DOI:** 10.3390/s25020565

**Published:** 2025-01-19

**Authors:** Jinhua Liu, Yongsheng Shi, Dongjin Huang, Jiantao Qu

**Affiliations:** 1 Shanghai Film Academy, Shanghai University, Shanghai 200072, China; jinhua1427@hotmail.com (J.L.); djhuang@shu.edu.cn (D.H.); hnzkqjt@shu.edu.cn (J.Q.); 2 Shanghai Engineering Research Center of Motion Picture Special Effects, Shanghai 200072, China

**Keywords:** endoscopic image, 3D reconstruction, neural radiance fields, soft tissue dynamics, image segmentation

## Abstract

The advancement of neural radiance fields (NeRFs) has facilitated the high-quality 3D reconstruction of complex scenes. However, for most NeRFs, reconstructing 3D tissues from endoscopy images poses significant challenges due to the occlusion of soft tissue regions by invalid pixels, deformations in soft tissue, and poor image quality, which severely limits their application in endoscopic scenarios. To address the above issues, we propose a novel framework to reconstruct high-fidelity soft tissue scenes from low-quality endoscopic images. We first construct an EndoTissue dataset of soft tissue regions in endoscopic images and fine-tune the Segment Anything Model (SAM) based on EndoTissue to obtain a potent segmentation network. Given a sequence of monocular endoscopic images, this segmentation network can quickly obtain the tissue mask images. Additionally, we incorporate tissue masks into a dynamic scene reconstruction method called Tensor4D to effectively guide the reconstruction of 3D deformable soft tissues. Finally, we propose adopting the image enhancement model EDAU-Net to improve the quality of the rendered views. The experimental results show that our method can effectively focus on the soft tissue regions in the image, achieving higher fidelity in detail and geometric structural integrity in reconstruction compared to state-of-the-art algorithms. Feedback from the user study indicates high participant scores for our method.

## 1. Introduction

Endoscopy is an essential tool in the diagnosis and treatment of gastrointestinal diseases [1]. However, monocular endoscopes based on 2D imaging may compromise surgical and diagnostic tasks due to the lack of depth perception and detailed spatial information. Detailed 3D surgical scene reconstruction technology with enhanced depth perception can not only improve the accuracy and quality of surgery, but also play an important role in downstream tasks such as medical education and training, and surgical planning. However, robust algorithms are severely lacking in medical environments, where capturing high-fidelity diseased tissue is of the utmost importance. Therefore, developing a new method for reconstructing deformable tissue scenes is a pivotal technological and intellectual challenge in the field of Computer-Aided Diagnosis (CAD).

In early research on medical image reconstruction, discrete representation methods (such as point clouds and grids) were often used to model and reconstruct three-dimensional structures. The advent of deep learning has shown great promise in many other fields. Researchers have attempted to directly apply deep learning methods to discrete 3D data representations and have developed end-to-end 3D depth reconstruction algorithms [2,3,4] based on point clouds and grids, which can be used for various types of medical imaging data. However, in endoscopic scenes, traditional 3D discrete representation methods struggle to reconstruct relatively complete geometric structures of scenes. With the great success of NeRFs in scene reconstruction using continuous representations [5,6,7], several research works are gradually introducing NeRF-based implicit modeling methods into the 3D reconstruction of endoscopic images. For example, the development of EndoNeRF [8] and Endosurf [9] has attracted widespread attention due to their significant progress in enhancing the accuracy and detail fidelity of endoscopic image reconstruction. However, these methods primarily handle stereo endoscopic image sequences with a fixed viewpoint, which limits their applicability to monocular endoscopes with varying perspectives.

**Figure 1:**
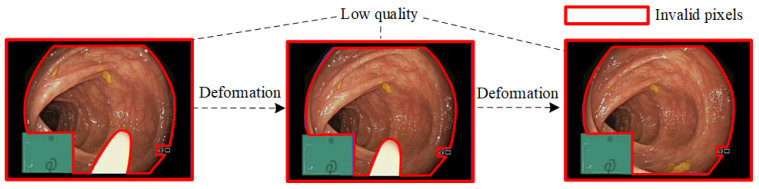
The challenge of monocular endoscopic image reconstruction. The regions marked by red lines in the figure are invalid pixels.

As shown in Figure 1, reconstructing high-fidelity deformable tissue from endoscopic images is a challenging and arduous task due to three key issues: First, the endoscopic images exhibit invalid occlusions caused by non-soft tissue regions, such as surgical tools, surrounding black and green pixels, and metadata information. The green pixels typically represent regions used for displaying navigation information or providing localization references during endoscopic surgeries. These pixels may overlay the endoscopic image to aid in guiding the surgeon. The surrounding black pixels arise from areas outside the endoscope’s field of view. These black areas are inherent to the imaging setup and do not provide useful information for 3D reconstruction. On the other hand, metadata refers to additional information overlaid on the image, such as real-time metrics (e.g., video bitrate, dropped frames) or textual annotations like video identifiers. Occlusion of invalid regions can interfere with the model’s reconstruction of geometric information, resulting in misalignment or artifacts in the reconstructed scenes. Manually drawing masks to accurately identify soft tissue regions is time-consuming and complex, increasing both the algorithm’s time cost and the operational burden on doctors. At present, there is no dataset specifically distinguishing between soft tissue regions and non-soft tissue regions (i.e., invalid regions). Second, soft tissues have non-rigid properties, and accurately capturing their dynamic deformation over time and with changing viewpoints is a critical challenge in reconstruction. Third, due to lighting limitations and the inherent characteristics of NeRFs, rendered endoscopic images often suffer from poor quality, including uneven lighting, low contrast, and blurry textures, which affect the reconstruction quality of endoscopic scenes.

To address the above issues, we propose a novel high-fidelity deformable soft tissue reconstruction framework based on neural radiance fields. First, to resolve the issue of invalid pixel occlusion in endoscopic images, we constructed a dataset specifically for segmenting soft tissue regions. Based on this dataset, we fine-tune SAM [10] and develop an enhanced version of the model, called SAM-EndoTissue. SAM-EndoTissue enables automatic and robust segmentation and generates soft tissue masks by excluding invalid regions from endoscopic images. Then, we propose a novel tissue mask-guided ray sampling strategy by incorporating tissue masks into the efficient dynamic neural radiance field, Tensor4D [7]. This strategy selectively reconstructs deformable tissue regions, while excluding invalid regions. Additionally, to address blurred textures in rendered views, we incorporate the previously proposed EDAU-Net (Encoder Dual Attention U-Net Network) to enhance the rendered views. The view optimization pipeline is seamlessly integrated with the reconstruction framework to build an end-to-end solution for high-fidelity reconstruction of 3D endoscopic scenes.

## 2. Related Work

Endoscopic imaging typically relies on discrete representation methods such as point clouds [2,3] and mesh grids [4]. These methods use sparse structures to efficiently generate surface models of simple scenes while utilizing additional warp fields to adapt to the dynamic changes of soft tissues. However, discrete representation methods face significant limitations and challenges in complex and dynamic endoscopic scenes. Endoscopic images captured within the human intestine often contain low-texture or no-texture areas that lack matching features. This sparse characteristic may lead to the loss of tissue details during the reconstruction process, thereby reducing reconstruction accuracy. In addition, discrete methods are difficult to adapt to irregular topological structures when dealing with dynamic soft tissues.

The continuous representation methods model 3D scenes through implicit or continuous functions, which can capture the fine details and complex dynamic structures in the scene. Neural radiance fields (NeRFs) [5] have become a typical representative of continuous representation methods due to their efficient reconstruction capability and excellent detail performance. With the development of NeRFs and their extensions [6,11], there is enormous potential for novel view generation and 3D reconstruction in dynamic real-world scenarios to support downstream applications such as telemedicine, surgical path planning and simulation, medical education, and surgical training. In medical image processing of CT scans or MRI, NeRFs can reconstruct complex anatomical structures with rich details, potentially reducing patients’ exposure to multiple high-level ionizing radiation or high-resolution scans [12]. For example, MedNeRF [13] and UMedNeRF [14] have successfully achieved the rendering of high-quality CT projections from a given few or even a single-view X-ray using a NeRF-based architecture. Iddrisu et al. [15] reconstructed the 3D geometry and appearance of brain structures from 2D brain MRI images, aiding doctors to more intuitively understand the morphology and location of the lesions. In X-ray image processing, Maas et al. [16] achieved 3D reconstruction of blood vessels from 2D X-ray angiography images with sparse views and limited angles. Although NeRFs have brought revolutionary progress to the reproduction of 3D scenes in CT, MRIs, and X-ray images, the above methods usually deal with static scenes. For endoscopic images, dynamic soft tissue structures need to be captured and reconstructed. Therefore, these methods still face certain limitations in the 3D reconstruction of endoscopic images.

Currently, research in this field focuses on adapting NeRFs to better fit the unique requirements of endoscopy data. Some research works have achieved significant success in reconstructing single-viewpoint stereoscopic endoscopic images by using variants of deformable radiation fields. For example, Wang et al. [8] proposed EndoNeRF, which first applied D-NeRF [6] to the 3D reconstruction of binocular endoscopic images, restoring high-quality details of surgical scenes. Considering the constant changes in surgical scenes caused by instrument movement, Endosurf [9] designed a novel NeRF-based method to reconstruct accurate 3D information from single-viewpoint RGBD images. These models are capable of reconstructing deformable tissue scenes within the human body. However, they are limited to input views with a single viewpoint and require paired binocular endoscopic image data to obtain stereo-depth information. Moreover, these methods are usually time-consuming during both training and rendering. EndoGaussian [17] utilized the emerging 3D Gaussian Splatting [11] to improve the efficiency of soft tissue reconstruction in stereoscopic endoscopic videos. However, this method relies on local data points to estimate the overall structure and requires high-quality data to reconstruct accurate 3D models effectively. Soft tissues with limited viewpoints and poor surface features may lead to significant holes or errors in the reconstructed images. Currently, there are few reports on the reconstruction of monocular endoscopic images. Recent advancements demonstrate that modified NeRFs can reconstruct 3D scenes with different objectives, including dynamic scene representation [7], modeling for sparse views [18,19,20], and optimizing the performance of scene reconstruction or rendering [21,22,23]. Due to the unique characteristics of endoscopic images, existing methods are not fully applicable to the 3D reconstruction of such images. Based on the framework of NeRFs, new reconstruction methods are designed for complex scenarios within human cavities and are expected to promote the further development of medical-assisted diagnosis technology.

## 3. Methods

**Figure 2:**
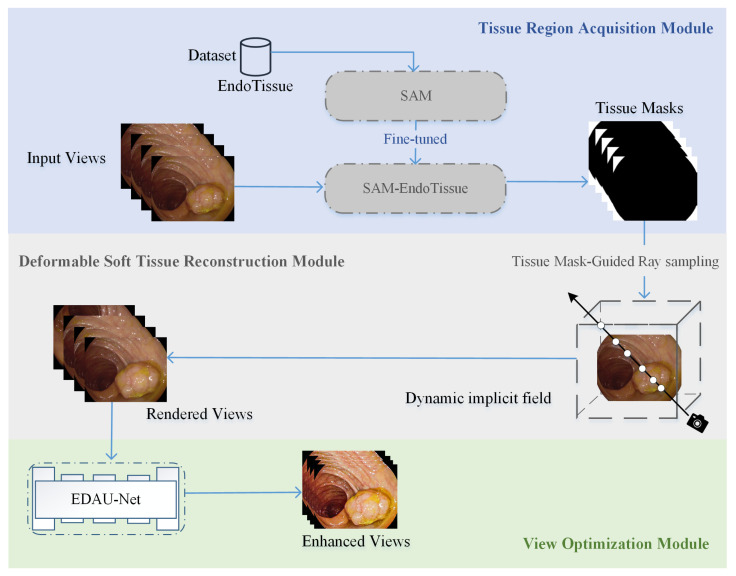
The framework of the proposed method.

Figure 2 shows the framework of our proposed method for high-fidelity deformable soft tissue reconstruction based on NeRFs, which consists of three main modules: tissue region acquisition, deformable soft tissue reconstruction, and view optimization.

(1) The tissue region acquisition module mainly utilizes the constructed dataset EndoTissue to fine-tune and retrain SAM to obtain an extended version, named SAM-EndoTissue. SAM-EndoTissue can quickly predict the tissue masks from input views, which directly contributes to the accuracy and efficiency of the deformable soft tissue reconstruction module.
(2) The deformable soft tissue reconstruction module uses tissue masks to perform tissue mask-guided ray sampling on Tensor4D to ensure that the trained NeRFs only reconstruct valid dynamic soft tissue scenes. This targeted sampling improves the accuracy and quality of 3D reconstruction in deformable endoscopic scenes.
(3) The view optimization module introduces the endoscopic image enhancement network EDAU-Net to enhance the rendered views of the dynamic implicit field. This module improves contrast, detail richness, and visual clarity, making the rendered images more interpretable and clinically applicable. Finally, we can obtain enhanced views.

### 3.1. Tissue Region Acquisition

Currently, there is no dataset used to segment soft tissue regions other than the invalid areas in endoscopic images. The SAM is a state-of-the-art segmentation model designed to segment objects in images with high precision. SAM performs automatic and interactive segmentation, guided by prompts such as points, boxes, or text, enabling flexible application to various scenarios. Unlike standard image processing techniques, SAM benefits from its rich feature representation, trained on a massive dataset of over 1 billion segmentation masks across diverse image domains, which provides robust generalization capabilities. Standard image processing methods can be sensitive to noise, lighting changes, and occlusions. These factors are common in endoscopic environments. SAM’s architecture is specifically designed to generalize well to such complex scenes, maintaining segmentation accuracy even under challenging conditions. SAM is robust and stable for complex scenarios, making it an ideal choice for segmenting specific regions in minimally invasive surgical environments. Moreover, SAM’s fully automatic segmentation mode effectively reduces labor costs. However, SAM is not suitable for most medical image segmentation tasks [24]. In order to train a deep learning network that can accurately and automatically segment soft tissues, this paper constructs the first dataset, EndoTissue, specifically designed for the segmentation of soft tissue regions from endoscopic images. Additionally, a simple fine-tuning strategy is designed to apply SAM to the segmentation task of soft tissue regions of medical images. This ensures that the method does not require additional operations by doctors during the surgical process, thereby avoiding any extra burden.

**Figure 3:**
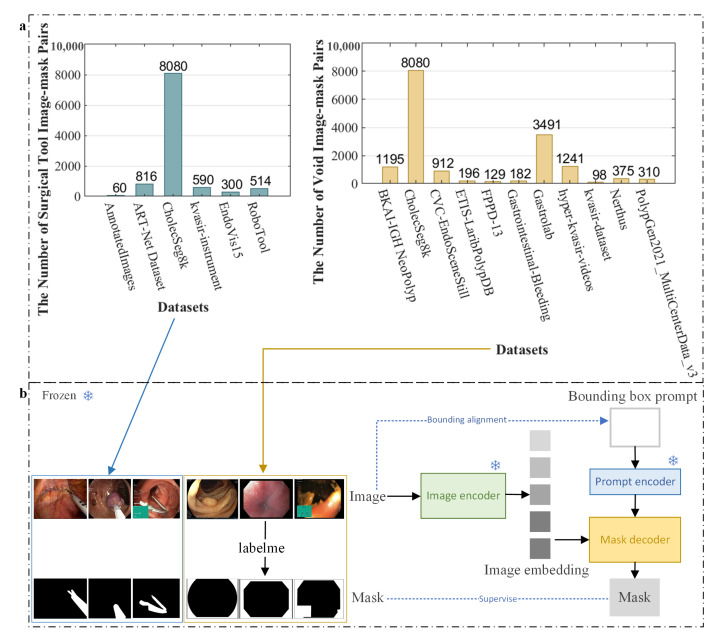
Endoscopic images and their mask images. (**a**) Source of endoscopic images for dataset EndoTissue. (**b**) The training process of fine-tuning SAM using the constructed dataset EndoTissue.

As illustrated in Figure 1, the invalid regions primarily consist of surgical tools, surrounding black and green areas, and metadata information. For surgical tools, some scholars have constructed datasets specifically for surgical tool segmentation. As shown in Figure 3a, the dataset includes 10,360 real endoscopic images and corresponding surgical tool segmentation masks (ground truth, GT) selected from the open-source medical imaging datasets: AnnotatedImages [25], ART-Net Dataset [26], CholecSeg8k [27], kvasir-instrument [28], EndoVis15 [29], and RoboTool [30]. The images in these datasets are all from endoscopic surgery scenes with invalid regions occluded by surgical tools. Therefore, we no longer need to manually annotate the regions where surgical tools are located. For the surrounding black and green regions, as well as metadata content, the dataset EndoTissue includes a total of 16209 real endoscopic images collected from the following open-source medical imaging dataset, including PolypGen [31,32,33], Nerthus [34], kvasir [35], Hyper-Kvasir [36], Gastrolab [37], Gastrointestinal-Bleeding [38], FPPD-13 [39], ETIS-LaribPolypDB [40], CVC-EndoSceneStill [41], CholecSeg8k [27], and BKAI-IGH NeoPolyp [42]. The images in these datasets are obscured by invalid pixels, such as large green areas and black regions surrounding the image. We invited four experts majoring in digital media technology from universities to manually annotate the soft tissue regions of these endoscopic images using labelme to obtain the corresponding segmentation masks (GT). All ground truths in the dataset EndoTissue are black-and-white binary images. Pixels in the soft tissue regions are set to black, while those in invalid regions are set to white. By observing the endoscopic images, we can see that the sequence images of each scene have the same invalid regions, meaning only one GT needs to be manually annotated by experts for each scene. The labeling process followed strict annotation guidelines, including detailed instructions for the precise labeling of soft tissue regions using the labelme tool and clear definitions of invalid areas in endoscopic images to ensure consistency and accuracy. Selecting images from multiple datasets with different scenes provides rich training samples for the segmentation of soft tissue regions, ensuring that the EndoTissue dataset can adapt to a wide range of medical image segmentation tasks. As shown in Figure 3b, the invalid regions in endoscopic images consist of two main types. The first type consists of areas occluded by surgical tools. The second type includes surrounding black and green regions as well as metadata information. These two types of datasets are used to fine-tune and retrain the SAM model, respectively, to improve the accuracy of segmenting soft tissue regions, resulting in two specialized models: SAM-EndoTissue1 and SAM-EndoTissue2 (for simplicity, they are collectively referred to as SAM-EndoTissue). To predict the region of interest (ROI) of endoscopic image sequences, we input the images into SAM-EndoTissue1 and SAM-EndoTissue2. The outputs from these two models are then merged to generate the final soft tissue masks that comprehensively represent the valid regions of the endoscopic images. SAM-EndoTissue not only removes black pixels but also excludes green areas, surgical tools, and metadata information, thereby achieving accurate reconstruction of valid soft tissue regions in endoscopic images. SAM-EndoTissue selects SAM’s fully automatic segmentation mode and fine-tunes the mask decoder, freezing the image encoder and prompt encoder. This mode does not require manually adding a bounding box for the selected ROI. Instead, the model automatically generates bounding box prompts of the same size as the original image to achieve segmentation. The fully automatic segmentation mode can improve the segmentation performance and reduce the cost of manual labeling. The tissue masks are automatically obtained by feeding the endoscopic images into the trained SAM-EndoTissue model.

### 3.2. Deformable Soft Tissue Reconstruction

**Figure 4:**
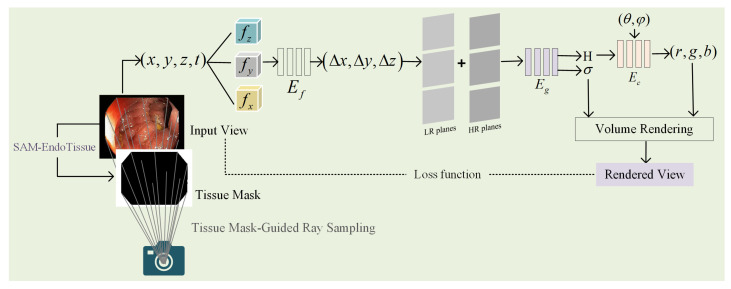
The overall flow of deformable soft tissue reconstruction.

The structures and morphologies in endoscopic image sequences usually change to varying degrees due to factors such as organ peristalsis, respiration, and changes in the force applied by surgical tools. This increases the challenge of reconstructing deformable tissues from endoscopic images. Tensor4D is an advanced method for reconstructing dynamic scenes efficiently and effectively. It is specifically designed to improve computational efficiency compared to the traditional NeRFs. It adopts 4D decomposition for D-NeRF in monocular dynamic scenarios, efficiently capturing the dynamic scenes inside the human body. By adopting hierarchical tri-projection decomposition, Tensor4D reduces memory consumption and achieves a compact representation of 4D spatio-temporal fields. The coarse-to-fine strategy also balances the efficiency of training and the quality of reconstruction, ensuring rapid convergence in the early training stages while preserving fine-grained details in the final output. However, occlusion of invalid regions may limit Tensor4D’s depth perception of objects in the scene. To further improve the accuracy of Tensor4D for endoscopic scene reconstruction, we propose a tissue mask-guided ray sampling strategy. This strategy enhances the reconstructed details and accuracy of the dynamic implicit field by focusing on the sampling of specific soft tissue regions in endoscopic images and further reduces computational overhead by avoiding unnecessary computations in invalid regions. The overall flow of deformable soft tissue reconstruction is shown in Figure 4.

**Scene Representation**: Tensor4D uses multilayer perceptron (MLP) network $E_{f}$, $E_{g}$, and $E_{c}$ to encode a dynamic 3D scene. For a point X=(x,y,z) in the scene at time *t*, we first decompose the 4D tensor into three feature volumes: $f_{z}$, $f_{y}$, and $f_{x}$. These feature volumes are further decomposed into nine flow feature planes, which are input into the flow MLP $E_{f}$ to predict the position offset ΔX=(Δx,Δy,Δz) from the starting time to the specified input time *t*. Then, the position offset is decomposed into three LR and HR feature planes. These feature planes and ΔX=(Δx,Δy,Δz) are fed into the geometry MLP $E_{g}$ to obtain the high-dimensional feature *H* and the volume density σ. Finally, the high-dimensional feature *H* and the perspective d=(θ,φ) are input into the color MLP $E_{c}$ to generate the reconstructed color c=(r,g,b).

**Tissue Mask-Guided Ray Sampling**: The ray casting of Tensor4D is applied to any pixel *p* in the entire image (i.e., the entire image scene participates in the reconstruction process), with the direction of the ray emitted from the camera center o passing through that pixel denoted as d. The point on the imaging ray corresponding to this pixel is defined as X(h)=o+d·h, where *h* represents the positional parameter of the sampling point along the ray. This method of directly reconstructing the entire image will affect the accuracy of reconstructing the region where the soft tissue is located. The binary mask can locate specific regions of the image, preventing them from participating in the subsequent reconstruction of implicit NeRFs [8]. To overcome the redundant sampling problem in the original Tensor4D reconstruction, this paper proposes a tissue mask-guided ray sampling strategy to optimize the ray sampling process of Tensor4D. Specifically, assuming *n* images *I* are input, we first cast rays from pixels randomly selected from these images. The tissue mask $I_{mask}=1$ acquired by the SAM-EndoTissue model can efficiently distinguish the soft tissue regions and invalid regions of the image. Based on the tissue mask, we determine whether a pixel is located in an invalid region or a valid soft tissue region. If $I_{mask}^{i}=0$, the i-th pixel ray is sampled. If $I_{mask}^{i}=1$, the i-th pixel ray is not sampled. In the ray casting process, each ray is defined as $X\left(h\right)=o+\omega^{\prime }\cdot d\cdot h$, where $\omega^{\prime }$ represents the weighting factors that guide the rays. The formula for $\omega^{\prime }$ is given below:

(1) $$\left\{\begin{array}{c} \omega^{\prime }=\frac{\omega }{{∥\omega ∥}_{F}}\\ \omega =\left(1+\frac{\sum_{i=1}^{n}I_{\mathrm{mask}\;}^{i}}{{∥\sum_{i=1}^{n}I_{\mathrm{mask}\;}^{i}∥}_{F}}\right)\times \left(1-I_{\mathrm{mask}\;}^{i}\right) \end{array}\right.$$

where ${∥•∥}_{F}$ represents the Frobenius normalization.

**Volume Rendering**: Tensor4D predicts the color value C(p,t) of the corresponding pixel *p* in the 2D image based on the color *c* and density σ of the sampled points in the scene at time *t* by using the volume rendering Equation (2):

(2) $$\begin{array}{c} C\left(p,t\right)=\int_{h_{n}}^{h_{f}}\tau \left(h,t\right)\sigma \left(p\left(h,t\right)\right)c\left(p\left(h,t\right),d\right)dh, \end{array}$$

(3) $$\begin{array}{c} \;\mathrm{where}\;p\left(h,t\right)=X\left(h\right)+\Psi_{\theta_{t}}\left(X\left(h\right),t\right), \end{array}$$

(4) $$\begin{array}{c} \left[c\left(p\left(h,t\right),d\right),\sigma \left(p\left(h,t\right)\right)\right]=\Psi_{\theta_{x}}\left(p\left(h,t\right),d\right), \end{array}$$

(5) $$\begin{array}{c} \;\mathrm{and}\;\tau \left(h,t\right)=\exp\left(-\int_{h_{n}}^{h}\sigma \left(p\left(s,t\right)\right)ds\right). \end{array}$$

where τ(h,t) is the cumulative transmittance of the ray from the nearest point $h_{n}$ to the farthest point $h_{f}$. p(h,t) represents the 3D point on the camera ray X(h) transformed to the canonical space using the deformation network $\Psi_{\theta_{t}}$.

**Network Training**: To train the dynamic NeRF network for the target scene, this paper uses three loss functions to evaluate the difference between the output and GT: feature regularization loss $L_{r}$, surface constraint loss $L_{e}$, and color loss $L_{rgb}$. The total loss function is expressed as follows:

(6) $$L_{m}=\lambda_{1}L_{r}+\lambda_{2}L_{e}+\lambda_{3}L_{rgb}$$

where the coefficients $\lambda_{1}$, $\lambda_{2}$, and $\lambda_{3}$ are set to 0.01, 0.2, and 1.0, respectively.

$L_{rgb}$ is used to calculate the mean squared error between the pixel values of the rendered image and those of the original image, thereby completing the training of the dynamic NeRFs for the target scene in a self-supervised manner. The loss function $L_{rgb}$ is formulated as follows:

(7) $$L_{rgb}=\frac{1}{N_{s}}\sum_{i=1}^{N_{s}}{∥C\left(p,t\right)-C_{gt}\left(p,t\right)∥}_{2}^{2}$$

where C(p,t) is the RGB color value of the image rendered by the canonical network. $C_{gt}\left(p,t\right)$ is the RGB color value of the input image. $N_{s}$ represents the set of sampling rays in the input view.

By minimizing the difference between adjacent elements in the same feature plane, $L_{r}$ can preserve the sparse structural characteristic of the feature plane. The equation is defined below:

(8) $$L_{r}=\sum_{T}\sum_{i,j}\sqrt{{\left(T_{i+1,j}-T_{i,j}\right)}^{2}+{\left(T_{i,j+1}-T_{i,j}\right)}^{2}}$$

$L_{e}$ can optimize the geometric representation by enhancing the smoothness of the surface. It can be represented by Equation (9):

(9) $$L_{e}={∥{∥\nabla_{S}\left(x,y,z,t\right)∥}_{2}-1∥}_{2}$$

where $\nabla_{S}$ denotes the gradient of the surface at position (x,y,z,t), which measures how much a surface varies in space.

### 3.3. Render View Optimization

**Figure 5:**
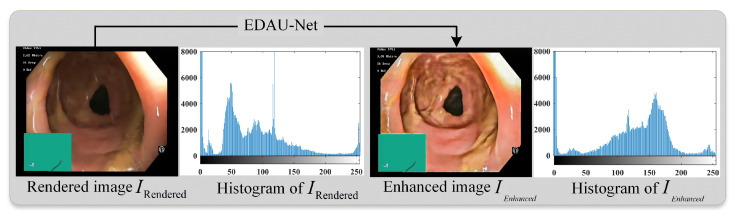
Effect comparison before and after the image enhancement model EDAU-Net.

Although the dynamic NeRFs learned in Section 3.2 can effectively reconstruct 3D scenes from endoscopic images, the input endoscopic images usually suffer from issues such as uneven illumination and low contrast due to the unique characteristics of the endoscopic imaging environment and equipment [43]. In addition, NeRFs rely on optimizing the shape and appearance of each 3D spatial location along a light ray solely based on individual pixel RGB values. This inherent limitation may result in the loss of edge details in the rendered views [44]. Therefore, images directly rendered using fully trained NeRFs can sometimes not meet the clinical needs. To further improve the fidelity of rendering views on the inner walls of human body cavities, this paper introduces our previous research work, the Encoder Dual Attention U-Net Network (EDAU-Net) [43], to enhance the rendered views. EDAU-Net is a novel deep learning-based global image enhancement network specifically designed to enhance endoscopic images. It improves the overall quality of the images by incorporating two innovative components into the U-Net framework: the Detail Attention Map module and the Luminance Attention Map module. The Detail Attention Map module is used to restore fine-grained details, while the Luminance Attention Map module is used to enhance the illumination of endoscopic images. EDAU-Net not only stably improves image texture and detail information but also effectively eliminates non-uniform luminance through supervised training on the large-scale endoscopic image enhancement dataset. The trained dynamic NeRFs generate the rendered image $I_{Rendered}$ by using the aforementioned volume rendering equation. Then, $I_{Rendered}$ is input into EDAU-Net to generate the enhanced image $I_{Enhanced}$:

(10) $$I_{Enhanced}=\mathrm{EDAU}\text{-}\mathrm{Net}\left(I_{Rendered};\theta \right)$$

where θ is the network parameter of EDAU-Net. Figure 5 illustrates the comparison results of rendering views before and after optimization. For the rendered views, the invalid regions excluded during the deformable soft tissue reconstruction phase are reintegrated into the image to provide a comprehensive representation of the surgical scene. After processing by EDAU-Net, the enhanced rendering view shows significant improvements in details, brightness, and contrast, while effectively suppressing noise and artifacts, as shown in Figure 5. Therefore, the view optimization module not only improves the quality of rendered endoscopic images, but also further optimizes the reconstruction performance of the dynamic NeRFs.

## 4. Results and Discussions

The experiments were performed in the following computing environment: Windows 10, CPU Intel(R) Xeon(R) E5-2620, CUDA 10.2, Python 3.7, PyTorch 1.5.0, and GPU Nvidia Titan Xp. In our implementation, we train our model using the Adam optimizer and set the iteration number to 200K. The batch size of rays is set to 512, and each is sampled 64 times along the ray.

**Figure 6:**
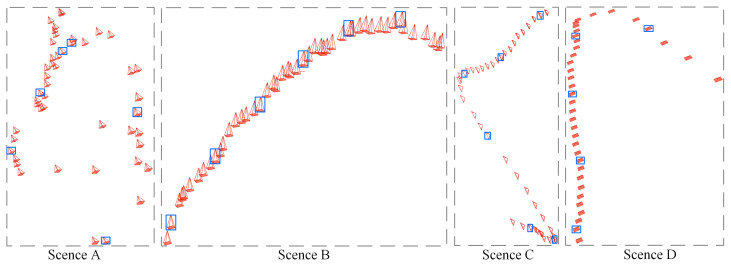
Camera positions of all scenes in 3D space. The blue boxes mark the cameras corresponding to the test images.

In this paper, we select four different scenes (A, B, C, and D) from the publicly available open-source endoscopic imaging datasets, the Nerthus dataset [34] and the Gastrolab Image Gallery [37], for qualitative and quantitative evaluation. Each scene has the characteristics of being obscured by invalid regions and dynamically changing in soft tissues. Moreover, each scene contains 37 to 48 sequential frames, with the size of either 720 × 576 or 640 × 480. Figure 6 illustrates the camera positions corresponding to the images of scenes A-D in 3D space obtained using COLMAP. In this figure, red markers represent the camera positions of the sequence frames used for training. The blue rectangular markers specifically highlight the camera positions of the sequence frames used for testing. We follow community standards [5] by holding out every eighth image as a test set for evaluating the effectiveness of the reconstruction. Additionally, we can observe the number of images captured from each scene and the spatial trajectory of the camera in Figure 6.

### 4.1. Qualitative Evaluation

To verify the effectiveness of our proposed method, we qualitatively compare it with state-of-the-art reconstruction methods, including NeRF [5], D-NeRF [6], DietNeRF [18], DS-NeRF [19], TiNeuVox [21], NRFF [22], 4D-GS [23], and Tensor4D [7].

**Figure 7:**
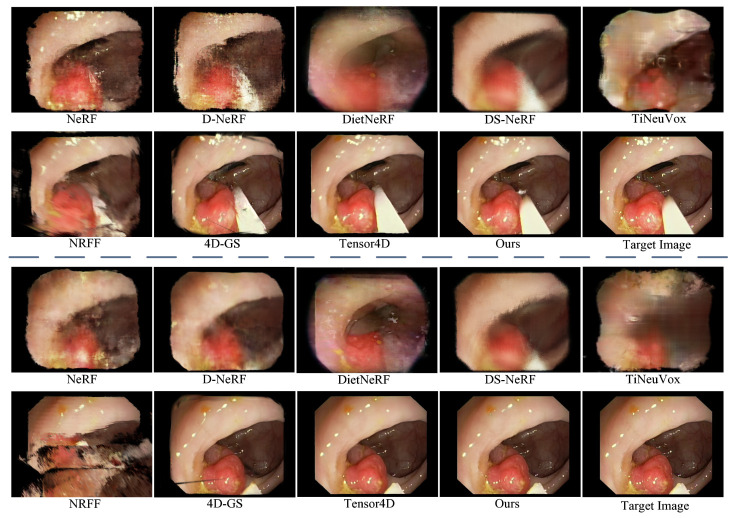
Visual comparison with state-of-the-art 3D reconstruction methods for the endoscopic image of Scene A.

Figure 7 shows the reconstruction results of various methods for Scene A at two different time points, which further verifies their performance in solving the problems of invalid region occlusion and soft tissue deformation in endoscopic images. The reconstruction results of NeRF reveal noticeable blurring and artifacts when dealing with complex dynamic soft tissue scenes. Similarly, D-NeRF exhibits prominent blurring and artifacts, with minimal improvement compared to NeRF. This is because D-NeRF relies on dense temporal sampling to learn the continuity of dynamic changes. In the endoscopy scenarios, the non-rigid deformation of soft tissues and the interference of invalid pixels make it difficult to accurately capture subtle dynamic changes. DietNeRF struggles to effectively handle the interference caused by black boundaries and surgical tools, resulting in multiple artifacts in the image and blurring in the soft tissue regions, making it difficult to accurately reconstruct the structures of the soft tissues. DS-NeRF incorporates depth information, which helps alleviate the issue of being obstructed by invalid regions to some extent. However, it still produces blurred structures and textures in the synthesized views when handling soft tissue deformations in the scene. TiNeuVox and NRFF struggle to handle the issue of being occluded by complex invalid pixels and cannot accurately reconstruct the geometric structure of the scene, resulting in significant misalignment. Although 4D-GS demonstrates some advantages in handling dynamic scenes, it exhibits a prominent “hollow” phenomenon in the synthesized views at both time points. This limitation arises because the 4D-GS model has sparse Gaussians in regions with few feature points. Tensor4D performs well in handling soft tissue deformation but still cannot avoid the loss of detailed information. In addition, 4D-GS and Tensor4D exhibit pixel misalignment across the rendered image, meaning that noticeable shifts in overall pixel positions are observed in some areas. Our proposed method significantly improves the reconstruction quality of the soft tissue regions. Meanwhile, the proposed method can capture the dynamic changes in the soft tissues and achieve high-fidelity reconstruction of dynamic scenes.

**Figure 8:**
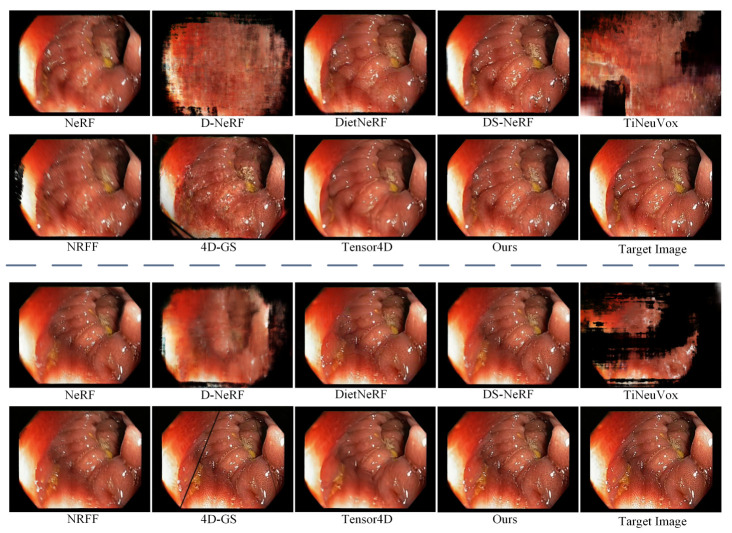
Visual comparison with state-of-the-art 3D reconstruction methods for the endoscopic image of Scene B.

Figure 8 shows the reconstructed results of our method and state-of-the-art comparative methods at different viewpoints in scene B. From Figure 8, we can observe that the soft tissue surfaces reconstructed by NeRF and NRFF exhibit varying degrees of blurring, indicating their limited adaptability to deformations. D-NeRF and TiNeuVox struggle to reconstruct the complete structural information of scene B, resulting in poor visual effects. 4D-GS still cannot reconstruct geometric structures with rich details due to its inherent limitations. The reconstructed soft tissue surfaces by DietNeRF and Tensor4D lack texture details. On the other hand, Tensor4D occasionally produces artifacts in regions undergoing rapid changes. Although DietNeRF performs relatively well in Scene B, it still has limitations in endoscopic scenes with non-rigid deformation of soft tissues and invalid pixel occlusion. Our proposed method achieves superior performance across all viewpoints compared with other reconstruction methods. Specifically, our method demonstrates significant advantages in the visual quality of rendered views, especially in generating texture details on the inner wall surface of the cavity. Moreover, our method can more accurately reconstruct the shape and appearance of the scene.

**Figure 9:**
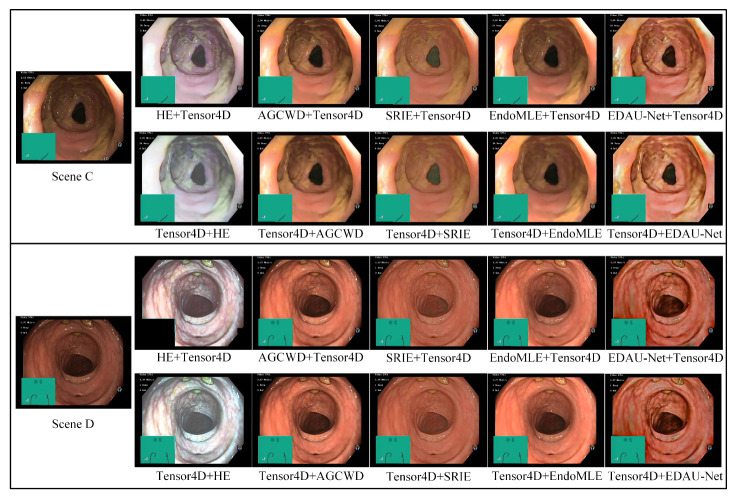
Visual comparison with state-of-the-art image enhancement methods for the endoscopic image of Scenes C and D.

To evaluate the effectiveness of EDAU-Net used in this paper, we compared EDAU-Net with classical 2D image enhancement methods, namely HE [45], AGCWD [46], SRIE [47], and EndoMLE [48]. We designed two experimental setups for comparison. In the first step, 2D enhancement methods were used to pre-process the dataset, followed by using Tensor4D to reconstruct endoscopic tissues, referred to as “* + Tensor4D”. In the second step, Tensor4D was used to reconstruct the endoscopic tissues, and then the 2D enhancement methods were used for post-processing the rendered views, named “Tensor4D + *”. All comparative experiments were conducted with the same parameters such as training epochs, batch size, and learning rate. The synthesized results of novel views for different 2D image enhancement methods are shown in Figure 9. From Figure 9, we observe that using image enhancement methods to process endoscopic images can improve the visual quality of images. Different enhancement methods have different applicability to endoscopic images. Notably, HE led to substantial color distortions in the images. SRIE improved the brightness of the image, but lost some detail information. AGCWD and EndoMLE improved the brightness of the image, but EndoMLE produced blurred textures. Compared with other image enhancement methods, EDAU-Net can significantly improve the brightness and contrast of endoscopic images, and obtain the clearest details visually.

### 4.2. Quantitative Evaluation

To comprehensively validate the performance of our proposed method, we used different evaluation metrics tailored to the specific goals of the comparisons.

To further validate that our proposed method has excellent reconstruction capability, we evaluated the quality of the rendered views of all methods using three widely used reconstruction image quality assessment metrics: Peak Signal-to-Noise Ratio (PSNR), Structural Similarity Index Measurement (SSIM), and Learned Perceptual Image Patch Similarity (LPIPS) [49]. PSNR measures the pixel-level fidelity between reconstructed images and target images, indicating the effect of noise reduction. A higher PSNR indicates better reconstruction accuracy, as it reflects a lower level of noise and distortion. SSIM is designed to mimic the human visual perception of image quality in the context of structural fidelity, where higher values (closer to 1) indicate greater similarity to the target image structure. LPIPS assesses perceptual differences between the reconstructed images and the target images by leveraging deep feature representations. Lower LPIPS values indicate higher perceptual similarity and better preservation of the original scene’s visual integrity. As shown in Table 1, the evaluation metric values of TiNeuVox and NRFF are significantly worse than those of other methods. This is because these methods of improving computational efficiency have a certain impact on the quality of the reconstructed scene, resulting in distortion and blurring of the reconstructed results. Among the compared methods, Tensor4D achieved the highest PSNR value of 26.3423 and SSIM value of 0.8152, and 4D-GS achieved the lowest LPIPS value of 0.2166 averaged across the test scenes A-D. In contrast, our proposed method outperformed all other methods with an average PSNR of 29.6029, SSIM of 0.8596, and LPIPS of 0.1866. Compared to the best-performing baseline methods, the proposed method achieved a 12.38% improvement in PSNR, a 5.44% improvement in SSIM, and a 13.86% reduction in LPIPS. This indicates that our method produces novel views with a higher degree of similarity to the GT, accurately reconstructing the intricate structures of soft tissues within the human body.

The real endoscopic images do not have corresponding high-quality images (i.e., GT) of the same scene. In order to effectively and objectively evaluate the performance of the EDAU-Net selected in this paper, three no-reference image quality assessment metrics specifically designed for enhancement tasks, namely Entropy, Contrast Improvement Index (CII), and Average Gradient (AG), were used to assess the quality of the enhanced views. Entropy quantifies the richness of image information, indicating the level of detail preserved or enhanced. CII measures the degree of contrast enhancement relative to the original image. AG reflects the sharpness and clarity of edges, which are critical factors for image enhancement. The comparison results are shown in Table 2. As observed, the performance of “* + Tensor4D” methods was generally inferior to that of “Tensor4D + *” methods. This may be because image enhancement techniques change the original color information and dynamic range of the image, which causes Tensor4D to learn the wrong scene information. In Scene C, Tensor4D + EDAU-Net outperformed all other methods, achieving improvements of 7.39% in Entropy, 4.89% in CII, and 1.30% in AG. In Scene D, Tensor4D + EDAU-Net demonstrated even greater gains, with improvements of 8.60% in Entropy and 29.66% in CII compared to the optimal values of these comparison methods. Although the AG was slightly lower than that of Tensor4D + HE in Scene D, the overall enhanced quality remained superior due to significant improvements in other metrics. Tensor4D + EDAU-Net achieved optimal comprehensive performance. Therefore, in this paper, we first improved Tensor4D to reconstruct the 3D scenes of endoscopic images, and then used EDAU-Net to post-process and enhance the rendered views.

### 4.3. Ablation Study

**Figure 10:**
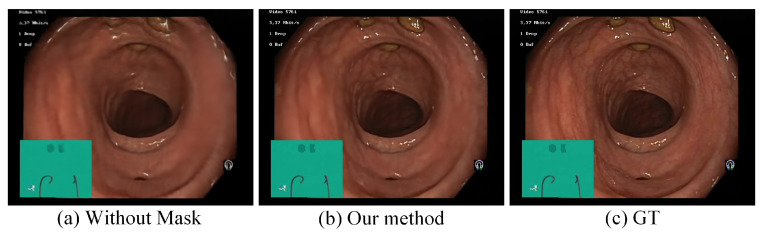
Ablation experiment results on tissue mask-guided ray sampling. (**a**) Without tissue mask-guided ray sampling; (**b**) our method; (**c**) ground truth.

The tissue mask-guided ray sampling strategy in Section 3.2 utilizes the masks predicted by the SAM-EndoTissue model to reconstruct the deformable soft tissue regions while preventing ray sampling of invalid regions in endoscopic images. To evaluate its impact, we conducted ablation experiments comparing the reconstructed results with and without this module. The reconstruction pipeline was initially executed by removing the tissue mask-guided ray sampling strategy, allowing rays to be sampled uniformly across the entire scene. Next, the same reconstruction pipeline was achieved using the tissue mask-guided ray sampling, restricting sampling to deformable soft tissue regions predicted by the SAM-EndoTissue model. The qualitative and quantitative results are shown in Figure 10 and Table 3. Figure 10a shows the reconstructed result after removing the tissue mask-guided ray sampling. Figure 10b is the reconstructed result by using the tissue mask-guided ray sampling. Figure 10b obviously has richer details and higher clarity than Figure 10a. Moreover, we can see that the PSNR, SSIM, and LPIPS metrics of the rendered view by adopting tissue mask-guided ray sampling are significantly improved, as shown in Table 3. This demonstrates that the quality and structural integrity of the generated images are enhanced.

**Figure 11:**
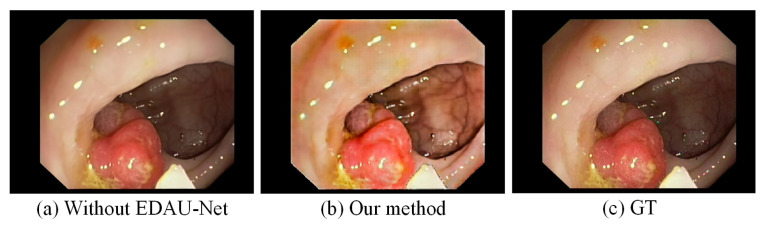
Ablation experiment results on EDAU-Net. (**a**) Without EDAU-Net; (**b**) our method; (**c**) ground truth.

In Section 3.3, the EDAU-Net is introduced to enhance the quality of rendered views. Its effectiveness was evaluated through an ablation study that compared the results with and without EDAU-Net processing. The rendered views generated by the reconstruction pipeline were directly compared in two scenarios: (1) without applying EDAU-Net, leaving the rendered views unprocessed, and (2) adopting the EDAU-Net to enhance the rendered views. Figure 11 shows the reconstructed results without and with EDAU-Net processing, respectively. Compared with Figure 11a,b, it can be seen that after EDAU-Net processing, the structure of the submucosal blood vessels in the dark region of the image is clearer, and the texture information is richer. Moreover, there is no over-enhancement problem in the highlight region. To objectively assess the quality of generated images before and after being processed by EDAU-Net, we use Entropy, AG, and Average Brightness (AB) to evaluate the effect of image enhancement. AB measures the mean pixel intensity of an image, providing an objective indication of overall brightness and illumination. Although higher AB values indicate improved brightness in underexposed regions, excessively high AB values can lead to visual discomfort and loss of detail due to overexposure. Typically, an AB value close to 128 (in the range of 0–255) is considered optimal. The quantitative results are displayed in Table 4. The values of Entropy and AG are significantly improved after using EDAU-Net as shown in Table 4. This indicates that EDAU-Net effectively improves the details and clarity of the images, making the generated images contain richer details. The proposed method improves AB values compared to the original images. This improvement improves the brightness of the images and effectively addresses underexposed regions, making them more visually interpretable.

### 4.4. User Study

**Figure 12:**
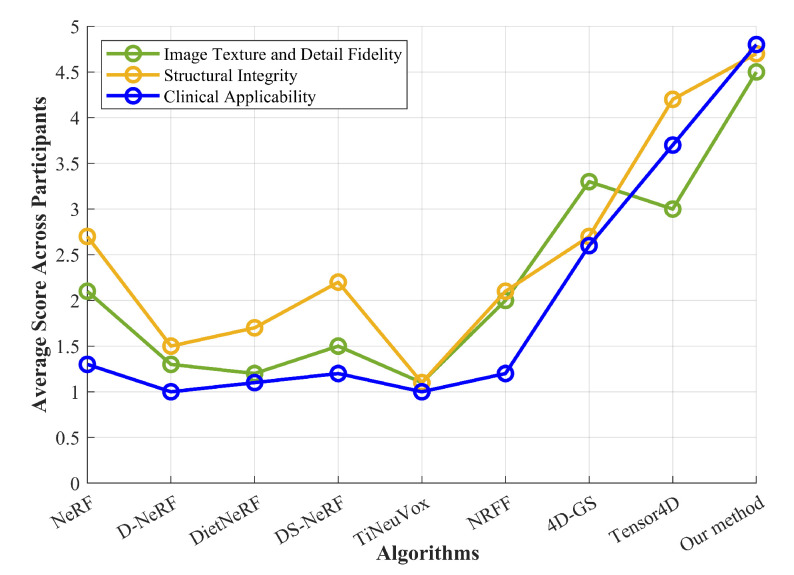
Results of user study.

We also conducted a user study involving two experienced clinicians and three senior medical students who had received training in endoscopy-related courses. They mainly evaluated the reconstructed results of NeRF, D-NeRF, DietNeRF, DS-NeRF, TiNeuVox, NRFF, 4D-GS, Tensor4D, and our proposed method on two randomly selected test images. To avoid bias, the images shown to participants were anonymized and the algorithms were typically labeled as Algorithm A, Algorithm B, etc. The experiment was conducted in a quiet and comfortable room to create an optimal environment for evaluation. All participants were assessed on the same high-resolution monitor, ensuring uniform viewing conditions across all sessions. To minimize potential distractions and allow for focused judgment, only one participant was present during each evaluation session. They took no less than 20 min each time. We used a Likert scale (1–5 points, where 1 indicates abysmal performance and 5 indicates excellent performance) and asked participants to rate the reconstructed results of these reconstruction algorithms based on image texture and the detail fidelity, structural integrity, and clinical applicability of the generated images. Before scoring, the three assessment criteria were briefly explained to these participants, and the output examples were presented to standardize their understanding of the scoring process. After completing the scoring, we immediately engaged each participant in a follow-up conversation to collect the qualitative feedback through open-ended questions. Each participant spent approximately 7–14 min participating in these conversations. Participants were encouraged to share specific thoughts on the strengths, weaknesses, and potential areas for improvement of these algorithms. Feedback was collected from five participants. The results of the user study are shown in Figure 12.

Figure 12 shows the average values of different test cases scored by different participants considering different aspects. As can be seen from Figure 12, both clinicians and medical students evaluated our proposed method as significantly superior to these comparative algorithms in terms of the fidelity of texture and details of the reconstructed images. Earlier proposed NeRFs such as NeRF and D-NeRF had lower scores. In particular, D-NeRF only scored 1.3 points, indicating that it performed poorly in terms of fidelity of textures and details. In terms of restoring structural integrity, our proposed method received the highest average score (4.7). Compared with other comparison methods, Tensor4D performed better (reaching 4.2 points), but it was still inferior to our reconstruction method. TiNeuVox performed poorly in terms of structural integrity, with an average score of only 1.1. In this paper, our proposed 3D reconstruction method received the highest average score in terms of clinical applicability. The evaluation of the participants shows the potential of our method to be applied in clinical settings. However, other comparative algorithms such as NeRF, D-NeRF, DietNeRF, DS-NeRF, TiNeuVox, and NRFF had lower scores, indicating that their clinical application value is limited. Observing the line graph, it can be seen that the overall values of these algorithms show a trend of gradually increasing. This is because with the continuous development of NeRFs, the effect of such methods on scene reconstruction is constantly improving.

In addition to the Likert scale ratings, we collected qualitative feedback from the five participants through open-ended questions. Their comments provided valuable insights into the clinical applicability and potential limitations of the proposed 3D reconstruction method. Several participants emphasized the potential advantages of the framework for surgical planning and diagnostics. For example, the second participant noted “The reconstruction models effectively capture structural integrity, which is critical for pre-surgical evaluation”. Similarly, the fourth participant commented that “The texture fidelity and spatial consistency of the reconstructed results are promising for training and educational purposes”. Furthermore, three participants, including experienced clinicians, emphasized the importance of optimizing both computational speed and system usability to enhance the framework’s feasibility for real-time clinical use. For example, the third participant highlighted that “A simplified user interface would be beneficial to improve workflow efficiency during routine clinical tasks”. The first participant raised a concern about “Whether the reconstruction speed could meet the demands of a live surgery scenario”. The fifth participant suggested that “Further optimization is needed to ensure the method can effectively reconstruct dynamic soft tissues”. This feedback highlights both the clinical potential of our method and the importance of addressing practical challenges. Feedback from participants provides valuable guidance for future research directions.

## 5. Conclusions and Future Works

In this paper, we constructed a dataset EndoTissue for soft tissue segmentation of endoscopic images for the first time. The EndoTissue dataset addresses the challenge of automatically segmenting soft tissue regions in endoscopic images, facilitating the effective and stable reconstruction of 3D scenes from endoscopic images. To achieve high-quality 3D reconstruction of deformable soft tissues in endoscopic images, a novel high-fidelity soft tissue reconstruction method is proposed in this paper. Our proposed method not only accounts for deformation factors but also introduces tissue mask-guided ray sampling and the EDAU-Net to improve the accuracy and stability of reconstruction, enabling the high-fidelity reconstruction of soft tissue structures from endoscopic image sequences. Experimental results show that compared with the state-of-the-art reconstruction methods, our proposed method has significant advantages in handling invalid region occlusion and dynamic changes in soft tissues in different scenes. It has also been highly praised by doctors and medical students. Therefore, the proposed method has important scientific research and application value in the field of endoscopic image processing.

In the future, we plan to focus on the automatic and accurate segmentation of lesion regions in reconstructed endoscopic scenes. We are committed to providing reliable solutions for identifying and analyzing pathological areas. Additionally, based on the feedback from participants, we will focus on optimizing computational pipelines and developing user-friendly interfaces. This exploration is expected to improve diagnostic accuracy while providing valuable support for preoperative planning and intraoperative decision-making.

## Figures and Tables

**Table 1:** PSNR, SSIM, and LPIPS metrics of each method on test images of Scenes A-D. ↑ means that the larger the value of the corresponding objective index, the better the reconstruction result. ↓ means that the smaller the value of the corresponding objective index, the better the reconstruction result.

Scenes	Metrics	NeRF [5]	D-NeRF [6]	DietNeRF [18]	DS-NeRF [19]	TiNeuVox [21]	NRFF [22]	4D-GS [23]	Tensor4D [7]	Our Method
Scene A	PSNR ↑	17.9609	20.4655	16.1259	21.0403	15.6641	16.8385	24.6080	28.0054	**31.4408**
	SSIM ↑	0.6875	0.7181	0.6223	0.6704	0.6253	0.6439	0.8582	0.8678	**0.9108**
	LPIPS ↓	0.4645	0.4436	0.5459	0.5712	0.5240	0.4119	0.1695	0.2034	**0.1221**
Scene B	PSNR ↑	25.2728	14.9028	23.5709	26.5093	10.4034	18.9101	23.0859	25.0928	**27.3171**
	SSIM ↑	0.7062	0.4070	0.6705	0.7399	0.2569	0.5941	0.7369	0.6850	**0.7507**
	LPIPS ↓	0.2842	0.6353	0.2468	0.2048	0.7028	0.3809	0.1726	0.3515	**0.1688**
Scene C	PSNR ↑	22.0996	22.9206	15.0100	21.5059	14.9503	20.5088	19.4483	25.2044	**28.4696**
	SSIM ↑	0.7902	0.7670	0.5479	0.6618	0.5396	0.7161	0.7640	0.8325	**0.8739**
	LPIPS ↓	0.2764	0.3545	0.5956	0.5729	0.5614	0.3202	0.2977	0.2742	**0.2557**
Scene D	PSNR ↑	25.9646	25.1803	18.9113	19.7004	17.7257	21.8376	22.9236	27.0666	**31.1840**
	SSIM ↑	0.8612	0.7998	0.6413	0.5165	0.5516	0.7104	0.8547	0.8755	**0.9029**
	LPIPS ↓	0.2074	0.3113	0.5100	0.7193	0.5513	0.3450	0.2266	0.2616	**0.1997**

Bold values denote the best performance value across all methods.

**Table 2:** Entropy, CII, and AG metrics of each method on test images of Scenes C and D. ↑ means that the larger the value of the corresponding objective index, the better the reconstruction result.

**Image Enhancement Methods + Tensor4D**						
**Method**	**Scene C**			**Scene D**		
	**Entropy ↑**	**CII ↑**	**AG ↑**	**Entropy ↑**	**CII ↑**	**AG ↑**
HE [45] + Tensor4D	5.7582	0.4599	2.0523	5.8502	0.6969	2.6216
AGCWD [46] + Tensor4D	5.9596	0.4922	1.8587	5.8409	0.6388	2.1458
SRIE [47] + Tensor4D	5.5899	0.3471	1.6020	5.0860	0.3381	1.5625
Wang [48] + Tensor4D	5.8431	0.4532	1.7864	5.5116	0.4263	1.7616
EDAU-Net [43] + Tensor4D	5.8211	0.6396	2.1708	5.5740	0.5308	2.0507
**Tensor4D + Image Enhancement Methods**						
**Method**	**Scene C**			**Scene D**		
	**Entropy ↑**	**CII ↑**	**AG ↑**	**Entropy ↑**	**CII ↑**	**AG ↑**
Tensor4D + HE [45]	6.0615	0.7951	2.6484	5.9550	0.8631	**3.5562**
Tensor4D + AGCWD [46]	6.0028	0.6930	2.0965	5.7180	0.7641	2.2183
Tensor4D + SRIE [47]	6.0309	0.6532	1.8780	5.5070	0.7346	1.8288
Tensor4D + Wang [48]	6.8084	0.6027	1.9921	6.5969	0.5398	1.9688
Tensor4D + EDAU-Net [43]	**7.3108**	**0.8340**	**2.6828**	**7.1648**	**1.1190**	3.2933

Bold values denote the best performance value across all methods.

**Table 3:** Objective index results in terms of PSNR, SSIM, and LPIPS across all scenes. ↑ means that the larger the value of the corresponding objective index, the better the reconstruction result. ↓ means that the smaller the value of the corresponding objective index, the better the reconstruction result.

Scenes	Model	PSNR ↑	SSIM ↑	LPIPS ↓
Scene A	Without mask	28.0054	0.8678	0.2034
	Our method	**31.4408**	**0.9108**	**0.1221**
Scene B	Without mask	25.0928	0.6850	0.3515
	Our method	**27.3171**	**0.7507**	**0.1688**
Scene C	Without mask	25.2044	0.8325	0.2742
	Our method	**28.4696**	**0.8739**	**0.2557**
Scene D	Without mask	27.0666	0.8755	0.2616
	Our method	**31.1840**	**0.9029**	**0.1997**

Bold values denote the best performance value across all methods.

**Table 4:** Objective index results in terms of Entropy, AG, and AB across all scenes. ↑ means that the larger the value of the corresponding objective index, the better the reconstruction result.

Scenes	Model	Entropy ↑	AG ↑	AB
Scene A	Without EDAU-Net	6.2674	1.7278	83.8247
	Our method	**6.3132**	**2.9253**	**97.9863**
Scene B	Without EDAU-Net	6.6485	2.3685	81.1413
	Our method	**7.3614**	**3.7225**	**103.9284**
Scene C	Without EDAU-Net	6.9906	1.6466	69.6059
	Our method	**7.3108**	**3.3874**	**103.1466**
Scene D	Without EDAU-Net	6.7120	1.7364	58.1496
	Our method	**7.1648**	**4.0675**	**82.9822**

Bold values denote the best performance value across all methods.

## Data Availability

Data are contained within the article.

## References

[1] Taş M., Yılmaz B. (2021). Super resolution convolutional neural network based pre-processing for automatic polyp detection in colonoscopy images. Comput. Electr. Eng..

[2] Beetz M., Banerjee A., Ossenberg-Engels J., Grau V. (2023). Multi-class point cloud completion networks for 3D cardiac anatomy reconstruction from cine magnetic resonance images. Med. Image Anal..

[3] Qi C.R., Su H., Mo K., Guibas L.J. Pointnet: Deep learning on point sets for 3d classification and segmentation. Proceedings of the IEEE Conference on Computer Vision and Pattern Recognition.

[4] Kong F., Wilson N., Shadden S. (2021). A deep-learning approach for direct whole-heart mesh reconstruction. Med. Image Anal..

[5] Mildenhall B., Srinivasan P.P., Tancik M., Barron J.T., Ramamoorthi R., Ng R., Vedaldi A., Bischof H., Brox T., Frahm J.M. (2020). NeRF: Representing Scenes as Neural Radiance Fields for View Synthesis. Proceedings of the Computer Vision—ECCV 2020.

[6] Pumarola A., Corona E., Pons-Moll G., Moreno-Noguer F. D-nerf: Neural radiance fields for dynamic scenes. Proceedings of the IEEE/CVF Conference on Computer Vision and Pattern Recognition.

[7] Shao R., Zheng Z., Tu H., Liu B., Zhang H., Liu Y. Tensor4d: Efficient neural 4d decomposition for high-fidelity dynamic reconstruction and rendering. Proceedings of the IEEE/CVF Conference on Computer Vision and Pattern Recognition.

[8] Wang Y., Long Y., Fan S.H., Dou Q. (2022). Neural rendering for stereo 3d reconstruction of deformable tissues in robotic surgery. Proceedings of the International Conference on Medical Image Computing and Computer-Assisted Intervention.

[9] Zha R., Cheng X., Li H., Harandi M., Ge Z. (2023). Endosurf: Neural surface reconstruction of deformable tissues with stereo endoscope videos. Proceedings of the International Conference on Medical Image Computing and Computer-Assisted Intervention.

[10] Kirillov A., Mintun E., Ravi N., Mao H., Rolland C., Gustafson L., Xiao T., Whitehead S., Berg A.C., Lo W.Y. Segment anything. Proceedings of the IEEE/CVF International Conference on Computer Vision.

[11] Kerbl B., Kopanas G., Leimkühler T., Drettakis G. (2023). 3D Gaussian Splatting for Real-Time Radiance Field Rendering. ACM Trans. Graph..

[12] Wang X., Hu S., Fan H., Zhu H., Li X. (2024). Neural Radiance Fields in Medical Imaging: Challenges and Next Steps. arXiv.

[13] Corona-Figueroa A., Frawley J., Bond-Taylor S., Bethapudi S., Shum H.P., Willcocks C.G. Mednerf: Medical neural radiance fields for reconstructing 3d-aware ct-projections from a single X-ray. Proceedings of the 2022 44th Annual International Conference of the IEEE Engineering in Medicine & Biology Society (EMBC).

[14] Hu J., Fan Q., Hu S., Lyu S., Wu X., Wang X. UMedNeRF: Uncertainty-aware single view volumetric rendering for medical neural radiance fields. Proceedings of the 2024 IEEE International Symposium on Biomedical Imaging (ISBI).

[15] Iddrisu K., Malec S., Crimi A. (2023). 3D reconstructions of brain from MRI scans using neural radiance fields. Proceedings of the International Conference on Artificial Intelligence and Soft Computing.

[16] Maas K.W., Pezzotti N., Vermeer A.J., Ruijters D., Vilanova A. Nerf for 3d reconstruction from x-ray angiography: Possibilities and limitations. Proceedings of the VCBM 2023: Eurographics Workshop on Visual Computing for Biology and Medicine.

[17] Liu Y., Li C., Yang C., Yuan Y. (2024). Endogaussian: Gaussian splatting for deformable surgical scene reconstruction. arXiv.

[18] Jain A., Tancik M., Abbeel P. Putting nerf on a diet: Semantically consistent few-shot view synthesis. Proceedings of the IEEE/CVF International Conference on Computer Vision.

[19] Deng K., Liu A., Zhu J.Y., Ramanan D. Depth-supervised nerf: Fewer views and faster training for free. Proceedings of the IEEE/CVF Conference on Computer Vision and Pattern Recognition.

[20] Wei Y., Liu S., Rao Y., Zhao W., Lu J., Zhou J. Nerfingmvs: Guided optimization of neural radiance fields for indoor multi-view stereo. Proceedings of the IEEE/CVF International Conference on Computer Vision.

[21] Fang J., Yi T., Wang X., Xie L., Zhang X., Liu W., Nießner M., Tian Q. Fast dynamic radiance fields with time-aware neural voxels. Proceedings of the SIGGRAPH Asia 2022 Conference Papers.

[22] Han K., Xiang W. Multiscale tensor decomposition and rendering equation encoding for view synthesis. Proceedings of the IEEE/CVF Conference on Computer Vision and Pattern Recognition.

[23] Wu G., Yi T., Fang J., Xie L., Zhang X., Wei W., Liu W., Tian Q., Wang X. 4d gaussian splatting for real-time dynamic scene rendering. Proceedings of the IEEE/CVF Conference on Computer Vision and Pattern Recognition.

[24] Ma J., He Y., Li F., Han L., You C., Wang B. (2024). Segment anything in medical images. Nat. Commun..

[25] Maier-Hein L., Mersmann S., Kondermann D., Bodenstedt S., Sanchez A., Stock C., Kenngott H.G., Eisenmann M., Speidel S. (2014). Can masses of non-experts train highly accurate image classifiers? A crowdsourcing approach to instrument segmentation in laparoscopic images. Proceedings of the Medical Image Computing and Computer-Assisted Intervention–MICCAI 2014: 17th International Conference.

[26] Hasan M.K., Calvet L., Rabbani N., Bartoli A. (2021). Detection, segmentation, and 3D pose estimation of surgical tools using convolutional neural networks and algebraic geometry. Med. Image Anal..

[27] Hong W.Y., Kao C.L., Kuo Y.H., Wang J.R., Chang W.L., Shih C.S. (2020). Cholecseg8k: A semantic segmentation dataset for laparoscopic cholecystectomy based on cholec80. arXiv.

[28] Jha D., Ali S., Emanuelsen K., Hicks S.A., Thambawita V., Garcia-Ceja E., Riegler M.A., de Lange T., Schmidt P.T., Johansen H.D. (2021). Kvasir-instrument: Diagnostic and therapeutic tool segmentation dataset in gastrointestinal endoscopy. Proceedings of the MultiMedia Modeling: 27th International Conference, MMM 2021.

[29] EndoVis15 Endovis Sub-Challenge: Instrument Segmentation and Tracking. https://endovissub-instrument.grand-challenge.org/.

[30] Garcia-Peraza-Herrera L.C., Fidon L., D’Ettorre C., Stoyanov D., Vercauteren T., Ourselin S. (2021). Image compositing for segmentation of surgical tools without manual annotations. IEEE Trans. Med. Imaging.

[31] Ali S., Jha D., Ghatwary N., Realdon S., Cannizzaro R., Salem O., Lamarque D., Daul C., Ånonsen K.V., Riegler M. (2021). PolypGen: A multi-center polyp detection and segmentation dataset for generalisability assessment. arXiv.

[32] Ali S., Ghatwary N., Jha D., Isik-Polat E., Polat G., Yang C., Li W., Galdran A., Ballester M.Á.G., Thambawita V. (2024). Assessing generalisability of deep learning-based polyp detection and segmentation methods through a computer vision challenge. Sci. Rep..

[33] Ali S., Dmitrieva M., Ghatwary N., Bano S., Polat G., Temizel A., Krenzer A., Hekalo A., Guo Y.B., Matuszewski B. (2021). Deep learning for detection and segmentation of artefact and disease instances in gastrointestinal endoscopy. Med. Image Anal..

[34] Pogorelov K., Randel K.R., de Lange T., Eskeland S.L., Griwodz C., Johansen D., Spampinato C., Taschwer M., Lux M., Schmidt P.T. Nerthus: A bowel preparation quality video dataset. Proceedings of the 8th ACM on Multimedia Systems Conference.

[35] Pogorelov K., Randel K.R., Griwodz C., Eskeland S.L., de Lange T., Johansen D., Spampinato C., Dang-Nguyen D.T., Lux M., Schmidt P.T. Kvasir: A multi-class image dataset for computer aided gastrointestinal disease detection. Proceedings of the 8th ACM on Multimedia Systems Conference.

[36] Borgli H., Thambawita V., Smedsrud P.H., Hicks S., Jha D., Eskeland S.L., Randel K.R., Pogorelov K., Lux M., Nguyen D.T.D. (2020). HyperKvasir, a comprehensive multi-class image and video dataset for gastrointestinal endoscopy. Sci. Data.

[37] Gastrolab The Gastrolab Image Gallery. http://www.gastrolab.net/index.htm.

[38] Khan A., Malik H. Gastrointestinal Bleeding WCE Images Dataset. 2023. https://data.mendeley.com/datasets/8pbbjf274w/1.

[39] Wang H., Zhu Y., Qin W., Zhang Y., Zhou P., Li Q., Wang S., Song Z. (2022). EndoBoost: A plug-and-play module for false positive suppression during computer-aided polyp detection in real-world colonoscopy (with dataset). arXiv.

[40] Silva J., Histace A., Romain O., Dray X., Granado B. (2014). Toward embedded detection of polyps in wce images for early diagnosis of colorectal cancer. Int. J. Comput. Assist. Radiol. Surg..

[41] Vázquez D., Bernal J., Sánchez F.J., Fernández-Esparrach G., López A.M., Romero A., Drozdzal M., Courville A. (2017). A benchmark for endoluminal scene segmentation of colonoscopy images. J. Healthc. Eng..

[42] Ngoc Lan P., An N.S., Hang D.V., Long D.V., Trung T.Q., Thuy N.T., Sang D.V. (2021). Neounet: Towards accurate colon polyp segmentation and neoplasm detection. Proceedings of the Advances in Visual Computing: 16th International Symposium, ISVC 2021.

[43] Huang D., Liu J., Shi Y., Li C., Tang W. (2023). Deep polyp image enhancement using region of interest with paired supervision. Comput. Biol. Med..

[44] Trevithick A., Yang B. (2020). Grf: Learning a general radiance field for 3d scene representation and rendering. arXiv.

[45] Castleman K.R. (1996). Digital Image Processing.

[46] Huang S.C., Cheng F.C., Chiu Y.S. (2012). Efficient contrast enhancement using adaptive gamma correction with weighting distribution. IEEE Trans. Image Process..

[47] Fu X., Zeng D., Huang Y., Zhang X.P., Ding X. A weighted variational model for simultaneous reflectance and illumination estimation. Proceedings of the IEEE Conference on Computer Vision and Pattern Recognition.

[48] Wang L., Wu B., Wang X., Zhu Q., Xu K. (2022). Endoscopic image luminance enhancement based on the inverse square law for illuminance and retinex. Int. J. Med Robot. Comput. Assist. Surg..

[49] An H., Khan J., Kim S., Choi J., Jung Y. (2024). The Adaption of Recent New Concepts in Neural Radiance Fields and Their Role for High-Fidelity Volume Reconstruction in Medical Images. Sensors.

